# Comparative Evaluation of Classic Mechanical and Digital Goldmann Applanation Tonometers

**DOI:** 10.3390/diagnostics15141813

**Published:** 2025-07-18

**Authors:** Assaf Kratz, Ronit Yagev, Avner Belkin, Mordechai Goldberg, Alon Zahavi, Ivan Goldberg, Ahed Imtirat

**Affiliations:** 1 Department of Ophthalmology, Soroka University Medical Center, Beer-Sheva 84101, Israel; 2 Faculty of Health Sciences, Ben-Gurion University of the Negev, Beer-Sheva 84101, Israel; 3 Department of Ophthalmology, Meir Medical Center, Kfar Saba 4428164, Israel; 4 The Sackler Faculty of Medicine, Tel Aviv University, Tel Aviv 39040, Israel; 5 Ophthalmology Department, The Eisenberg R&D Authority, Shaare Zedek Medical Center, Jerusalem 9103102, Israel; 6 Department of Ophthalmology and Laboratory of Eye Research, Felsenstein Medical Research Center, Rabin Medical Center—Beilinson Hospital, Petach Tikva 49100, Israel; 7 Glaucoma Unit, Sydney Eye Hospital, Sydney, NSW 2001, Australia; 8 Discipline of Ophthalmology, Sydney University, Sydney, NSW 2745, Australia; 9 Eye Associates, Sydney, NSW 2000, Australia

**Keywords:** intraocular pressure, applanation tonometry, Goldmann tonometer, digital tonometry, device comparison

## Abstract

**Objectives**: The objective of this study was to evaluate the agreement and clinical interchangeability of intraocular pressure (IOP) measurements obtained with the mechanical Haag-Streit AT900 Goldmann applanation tonometer (mGAT) and the digital Huvitz HT5000 applanation tonometer (dGAT). **Methods**: This retrospective comparative study included 53 eyes of 28 patients undergoing routine ophthalmologic evaluation. Each eye underwent IOP measurement using both mGAT and dGAT in a randomized sequence. Central corneal thickness (CCT) was also recorded. Pearson’s correlation coefficient was used to determine correlation between paired IOP measurements. Bland–Altman plots were graphed for the analysis of differences for IOP between the instruments. **Results**: A total of 53 eyes of 28 patients (15 males) were included in the study. The mean age of the patients was 62.6 years. The mean mGAT and dGAT measurements were 16.3 ± 6.6 mmHg (range 9–50) and 16.4 ± 6.2 mmHg (range 8.8–45.9), respectively (*p* = 0.53). A strong, significant positive correlation was found for paired IOP measurements by the two instruments (r = 0.98; *p* < 0.0001). Bland–Altman analysis revealed 95% limits of agreement from −2.5 to +2.3 mmHg, with a small but statistically significant proportional bias favoring mGAT at higher IOP levels. Additionally, 91% of paired measurements were within ±2 mmHg. CCT-related differences were statistically and clinically insignificant. **Conclusions**: IOP measurements obtained with mGAT and dGAT were highly correlated and clinically interchangeable for the range tested. The Huvitz HT5000 may serve as a reliable alternative to the classic Goldmann tonometer in routine clinical settings.

## 1. Introduction

Intraocular pressure (IOP) measurement remains a fundamental component in the diagnosis, monitoring, and treatment of glaucoma, a chronic and progressive optic neuropathy that represents a major global cause of irreversible blindness. The timely detection and accurate assessment of IOP are crucial, as it is the most significant modifiable risk factor for glaucomatous damage. For this reason, IOP evaluation is routinely included in comprehensive ophthalmic examinations and is critical for assessing disease progression and treatment efficacy.

Over the decades, numerous technologies and methods have been developed to measure intraocular pressure (IOP), reflecting the clinical importance of accurate and reliable tonometry. These include a wide range of contact and non-contact devices such as pneumotonometry, rebound tonometry (iCare), non-contact tonometry (NCT or “air puff”), Tono-Pen applanation tonometry, dynamic contour tonometry (Pascal), transpalpebral tonometry (e.g., Diaton), and ocular response analyzers (ORA), each with unique mechanisms, advantages, and limitations. Despite the growing diversity and sophistication of these alternatives, Goldmann applanation tonometry (GAT), originally introduced by Goldmann and Schmidt in 1957, has withstood the test of time and remains widely regarded as the clinical gold standard for IOP measurement [1,2,3,4,5,6,7,8,9,10,11,12,13,14,15,16,17,18,19,20,21]. Its well-established reliability, reproducibility, and extensive validation in both clinical and research settings have made it the benchmark against which newer tonometry methods are compared. GAT’s consistent performance and central role in major glaucoma studies continue to support its prominence in ophthalmic practice.

In recent years, technological advances have led to the development of digital adaptations of the classic GAT, aiming to modernize IOP measurement while maintaining clinical accuracy. The first digital version of the traditional Goldmann tonometer, and to date still one of the very few, is the AT900D, introduced in the early 2010s by Haag-Streit (Köniz, Switzerland). This model features a digital backlit readout and improved visualization while preserving the mechanical design and user experience of the original device.

Several studies have evaluated the AT900D and confirmed its high level of accuracy and reproducibility. Comparative analyses between the AT900D and the classic mechanical Goldmann tonometer have consistently shown excellent agreement in IOP measurements, with strong inter-device correlation and low variability, supporting its clinical reliability and the possibility of routine use as a digital alternative [22,23,24,25].

Despite differences in display and data interface, both the AT900D and its mechanical predecessor are based on the same underlying physical principle—the Imbert–Fick law [26]. This law, which relates the internal pressure of a sphere to the external force required to flatten its surface, remains the cornerstone of applanation tonometry and ensures methodological continuity across both device types.

Following the introduction of the AT900D, the Huvitz HT5000 (Huvitz, Dongan-gu, Anyang-si, Gyeonggi-do, Republic of Korea) emerged as an additional digital Goldmann-type applanation tonometer. Like the AT900D and the classic mechanical GAT, it is based on the Imbert–Fick principle. A key differentiator of the HT5000 is its substantially lower cost compared to the AT900D, making it a practical choice for ophthalmic clinics aiming to modernize equipment on a limited budget.

Despite being commercially available for over a decade, to the best of our knowledge, no peer-reviewed study has yet directly compared IOP measurements obtained with the Huvitz HT5000 and those of the gold-standard mechanical GAT. This notable gap in the literature raises important clinical questions about the interchangeability and reliability of the HT5000.

The aim of the present study was therefore to compare the performance of the Huvitz HT5000 digital applanation tonometer (dGAT) with that of the conventional Haag-Streit AT900 mechanical GAT (mGAT), focusing on the level of agreement, correlation, and potential differences across various IOP ranges. By analyzing the relationship between the two instruments, this study seeks to clarify whether this digital device can serve as a reliable substitute for the mechanical GAT in routine ophthalmologic settings.

## 2. Materials and Methods

This retrospective comparative study was conducted at a tertiary ophthalmology center and was based on a chart review of patients who underwent routine comprehensive ocular examinations. Eligible records were identified from the clinic database over a defined study period and included adult patients (aged ≥ 18 years) who had undergone IOP measurement using both mGAT and dGAT, as part of standard clinical care.

Inclusion criteria required complete and reliable IOP readings with both devices, along with a recorded measurement of central corneal thickness (CCT). Eyes with known corneal pathology (e.g., corneal scarring, dystrophies, edema, or prior refractive surgery) that could influence applanation accuracy were excluded. When both eyes met the inclusion criteria, they were included in the analysis.

Sample size estimation was based on previously published epidemiological data, which report an average IOP of approximately 15 mmHg with a standard deviation of 4 mmHg in populations similar to ours [27]. With an alpha level (α) of 0.05, a power (1 − β) of 0.80, and an anticipated clinically relevant difference of 1.6 mmHg, the minimum required sample size was calculated to be 49 eyes. To ensure adequate statistical power and account for possible exclusions, data from 53 eyes were ultimately included.

All measurements had been previously performed by a single experienced glaucoma specialist (AK) as part of routine care. Each patient had undergone full ophthalmic examination, including best-corrected visual acuity assessment, slit-lamp biomicroscopy, gonioscopy, indirect ophthalmoscopy, and pachymetry using ultrasound for CCT determination.

Intraocular pressure was measured using both the Haag-Streit AT900 mechanical Goldmann applanation tonometer (mGAT; Haag-Streit, Bern, Switzerland) and the Huvitz HT5000 digital Goldmann tonometer (dGAT; Huvitz, Dongan-gu, Anyang-si, Gyeonggi-do, Republic of Korea). Devices were calibrated regularly in accordance with manufacturer recommendations. The order of tonometry (mGAT vs. dGAT) had been randomly assigned at the time of the clinical visit to minimize measurement order bias. IOP was measured twice with each device per eye, and the average value was used for analysis.

To minimize the influence of repeated applanation on IOP measurements, a five-minute interval was observed between the two measurements [28]. To preserve examiner masking, the LED readout on the dGAT and the dial on the mGAT were concealed during measurement, and readings were recorded by an assistant. mGAT readings were rounded to the nearest whole mmHg in accordance with standard clinical conventions (even numbers when near a gauge mark; odd when between marks).

The reproducibility of each tonometer’s readings was assessed using the coefficient of variation (CV) from the paired measurements. Statistical analyses were performed using MedCalc version 20.110 (MedCalc Software, Mariakerke, Belgium). Paired Student’s t-tests were used to compare IOP means between devices, while Pearson’s correlation coefficient evaluated the linear relationship between paired readings. Agreement was assessed via Bland–Altman analysis. Linear regression models were employed to examine the association between inter-device IOP differences and CCT.

The study protocol was reviewed and approved by the institutional ethics committee and adhered to the principles of the Declaration of Helsinki (as revised in 2013).

## 3. Results

### 3.1. Demographics, IOP Results and Reproducibility

A total of 53 eyes from 28 patients were included in the final analysis. An initial review identified 56 eligible eyes, but three were excluded: one due to documented corneal irregularities that could interfere with applanation tonometry, and two due to insufficient patient cooperation during IOP measurement, which rendered the data unreliable.

Demographic characteristics and the distribution of ocular comorbidities are summarized in Table 1. The mean age of patients was 62.6 ± 21.3 years, and 15 (54%) were male. The mean central corneal thickness (CCT) was 549 ± 48 µm, and mean visual acuity was 0.3 ± 0.1 LogMAR. Common ocular conditions included cataract (in 35 eyes), glaucoma (25 eyes), and age-related macular degeneration (dry form: 13 eyes; wet form: 5 eyes).

Mean intraocular pressure (IOP) values obtained with mGAT and the digital dGAT were 16.3 ± 6.6 mmHg (range: 9–50 mmHg) and 16.4 ± 6.2 mmHg (range: 8.8–45.9 mmHg), respectively. The difference between these mean values was not statistically significant (*p* = 0.53), suggesting a high level of agreement between the devices across the studied population.

To evaluate intra-device reproducibility, the coefficient of variation (CV) was calculated based on the paired IOP readings obtained with each tonometer. The CV for the mGAT was 3.97% (95% confidence interval [CI]: 3.36–4.49%), while the CV for the dGAT was 4.59% (95% CI: 3.95–5.15%). No statistically significant difference was observed between the CVs of the two devices (*p* = 0.299), indicating comparable measurement repeatability under routine clinical conditions.

### 3.2. Correlation and Analysis of Differences Between Measurements

**Figure 1:**
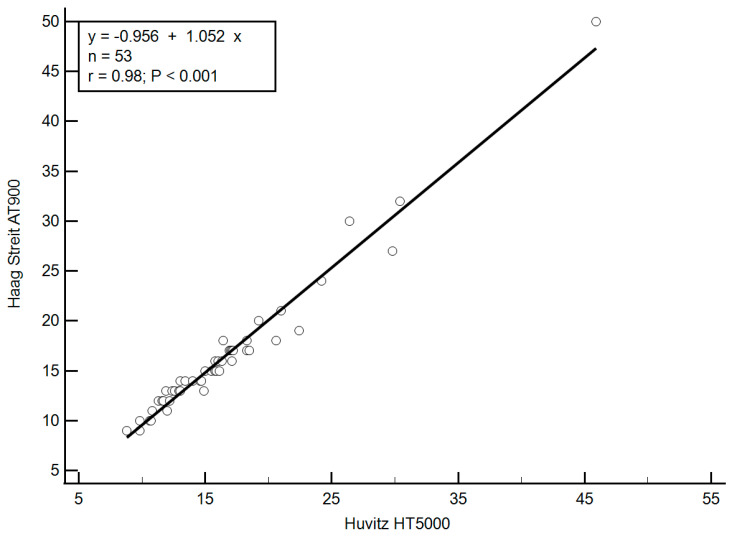
Correlation between IOP measurements (mmHg) obtained using mGAT and dGAT. A strong, statistically significant linear correlation was observed (r = 0.98, *p* < 0.0001). (IOP: Intra ocular pressure; mGAT: Haag-Streit AT900 mechanical Goldmann tonometer; dGAT: Huvitz HT5000 digital Goldmann tonometer).

A strong and statistically significant correlation was observed between IOP values measured by the mGAT and dGAT. The Pearson correlation coefficient was r = 0.98 (*p* < 0.001), indicating excellent agreement between the two devices across the entire IOP range. This correlation is illustrated in Figure 1.

In terms of absolute clinical agreement, 91% of all paired measurements fell within ±2 mmHg, and 96% were within ±3 mmHg, suggesting that in most cases, the difference between instruments was within a clinically acceptable margin (Table 2).

**Figure 2:**
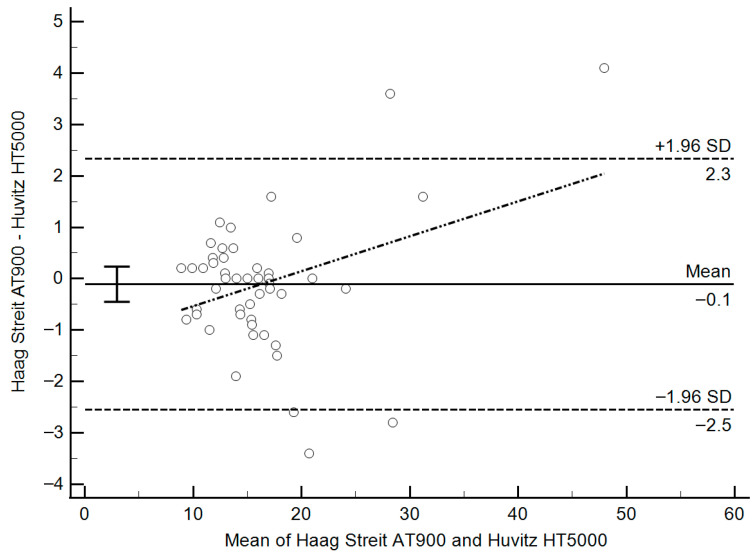
Bland–Altman analysis comparing IOP measurements from mGAT and dGAT (mmHg). The mean difference (bias) is centered near zero, with 95% limits of agreement from −2.5 mmHg to +2.3 mmHg. A weak but statistically significant proportional bias was detected (r = 0.35, *p* = 0.011), suggesting a tendency for mGAT to yield higher readings at higher IOP levels. (IOP: Intra ocular pressure; mGAT: Haag-Streit AT900 mechanical Goldmann tonometer; dGAT: Huvitz HT5000 digital Goldmann tonometer).

To further evaluate inter-device agreement, a Bland–Altman plot was constructed by plotting the difference between mGAT and dGAT measurements against the average of the two readings (Figure 2). The 95% limits of agreement ranged from −2.5 mmHg to +2.3 mmHg, reflecting the distribution of differences. The plot revealed a weak but statistically significant proportional bias, as demonstrated by a positive correlation between the average IOP and the inter-device difference (r = 0.35, *p* = 0.011). This indicates that the difference in IOP readings increased slightly with higher IOP values.

A linear regression analysis was conducted to quantify this bias. The regression equation was as follows: Difference = (0.0679 × mean IOP) − 1.216. Based on this model, an “equilibrium point” of 17.9 mmHg was identified—the value at which both instruments were expected to yield the same IOP reading. For IOP values above 17.9 mmHg, mGAT tended to report slightly higher IOP than dGAT, increasing by approximately 0.0679 mmHg for every 1 mmHg increase in pressure. Conversely, below this point, mGAT reported slightly lower IOP values compared to dGAT.

**Figure 3:**
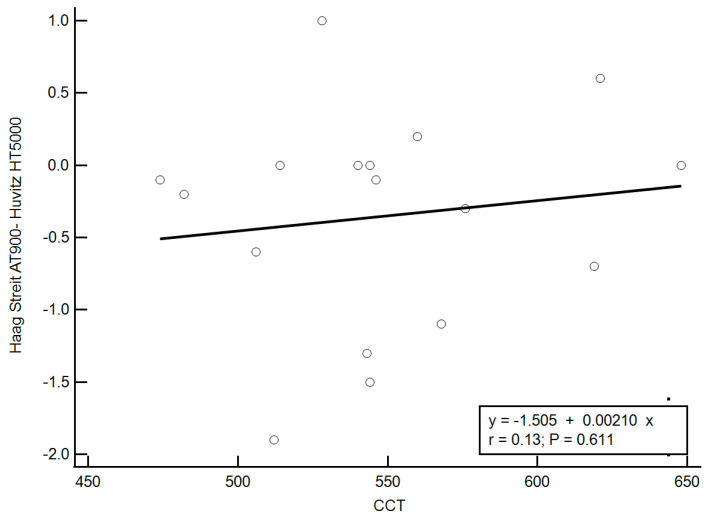
Relationship between CCT (µm) and the difference in IOP measurements (mGAT − dGAT, mmHg). A weak, non-significant positive correlation was observed (r = 0.13, *p* = 0.61), with minimal influence of CCT on inter-device variability. Regression equation: Difference = (0.0021 × CCT) − 1.505. (IOP: Intra ocular pressure; mGAT: Haag-Streit AT900 mechanical Goldmann tonometer; dGAT: Huvitz HT5000 digital Goldmann tonometer; CCT: Central corneal thickness).

The potential influence of central corneal thickness (CCT) on the discrepancy between instruments was also evaluated. A weak, non-significant positive correlation was found between CCT and the difference in IOP readings (r = 0.13, *p* = 0.61), as shown in Figure 3. The regression equation describing this relationship was: Difference = (0.0021 × CCT) − 1.505. This model suggests that mGAT tended to yield higher IOP readings than dGAT in eyes with thicker corneas, and vice versa for thinner corneas. The theoretical crossover, or equilibrium point, occurred at a CCT value of 717 µm. For each additional 1 µm above this point, the mGAT was expected to read approximately 0.0021 mmHg higher than the dGAT, while for each µm below this threshold, mGAT readings would be lower by the same magnitude.

## 4. Discussion

GAT remains the gold standard for IOP measurement due to its long-standing clinical validation, reproducibility, and widespread use [1,2,3,4,5,6,7,8,9,10,11,12,13,14,15,16,17,18,19,20,21]. However, it is not without limitations. Both mechanical and digital GAT instruments operate according to the Imbert–Fick principle [26], which assumes that the eye behaves as a thin-walled sphere with a perfectly flexible and dry surface—conditions not met in vivo. As a result, GAT is affected by several physiological and technical factors including CCT, corneal curvature, corneal hysteresis, the volume and concentration of fluorescein used, the impact of consecutive measurements, accommodation, and even examiner viewing angle [5,12,29,30,31,32]. These sources of error are equally applicable to digital tonometry platforms that retain the same underlying physical principle, such as the Huvitz HT5000 and the Haag-Streit AT900D.

Digital GAT instruments offer several practical advantages. One major improvement is the ability to display IOP values in increments of 0.1 mmHg, in contrast to the 2 mmHg scale divisions on traditional mechanical devices. This refinement eliminates the well-documented “hedgehog effect”—the digit preference toward even-numbered readings observed in mechanical tonometry [33,34]. Nevertheless, while the increased numerical resolution may convey an impression of superior precision, clinicians should remain mindful that the inherent accuracy of applanation tonometry is still limited to approximately ±1.5 mmHg [4,5,13,14,15,35,36].

Other benefits of digital GAT devices include improved display visibility in low-light conditions, easier readability, and digital data recording capabilities.

However, these enhancements come with trade-offs. Digital tonometers such as the HT5000 and AT900D have a narrower measurable IOP range (3–75 mmHg), as compared to the 0–80 mmHg range of mechanical GAT [37,38]. In clinical practice, this means that IOP readings below 3 mmHg or above 75 mmHg are displayed as “Lo” or “Hi,” respectively. While measurements above 75 mmHg are seldom required in routine ophthalmic practice, accurate assessment of very low IOP is essential in specific clinical scenarios, particularly in the early postoperative period following glaucoma filtration surgery. In such cases, differentiating between an IOP of 3 mmHg, 1 mmHg, or even 0 mmHg may carry significant therapeutic implications. Digital applanation tonometers, which may lack sufficient accuracy in the low-pressure range, are therefore not suitable for use in these critical postoperative settings.

Another practical limitation of digital applanation tonometers is their dependence on battery power (four AAA batteries per unit). In high-volume clinical settings, this may necessitate more frequent battery replacements, potentially resulting in increased operational costs and workflow disruptions, particularly if battery depletion occurs during a patient examination. Although these events are uncommon, they highlight a logistical consideration that distinguishes digital tonometers from their mechanical counterparts.

As with the classic mechanical Goldmann tonometer, the accuracy and routine calibration of digital applanation devices are essential for obtaining reliable IOP measurements. Both the Haag-Streit AT900 and the Huvitz HT-5000 utilize manufacturer-supplied calibration weights for checking device accuracy at two reference points: 20 mmHg and 60 mmHg. Calibration is verified by applying the appropriate weight and confirming the corresponding IOP reading. According to manufacturer recommendations, calibration checks should be performed monthly [37,38]. A deviation exceeding ±0.5 mmHg at 20 mmHg or ±1.0 mmHg at 60 mmHg is considered out of tolerance and requires recalibration or servicing. These shared calibration standards underscore the similar maintenance and quality assurance protocols required for both mechanical and digital Goldmann-based tonometers.

In the present study, IOP values measured using the mechanical GAT and the digital HT5000 showed excellent agreement. The mean difference between the two instruments was only 0.11 mmHg. Bland–Altman analysis demonstrated narrow 95% limits of agreement (−2.5 to +2.3 mmHg), and 91% of paired measurements were within ±2 mmHg, a difference considered clinically insignificant by most standards.

A weak but statistically significant proportional bias was identified, wherein the mGAT tended to report slightly higher IOP values at higher IOP ranges, with a crossover or “equilibrium point” at 17.9 mmHg. For example, at IOP levels of 30 or 40 mmHg, the HT5000 would read approximately 0.8 and 1.5 mmHg lower than the mechanical GAT, respectively. However, these deviations remain well within the expected variability range of applanation techniques and are unlikely to influence clinical decision-making in most cases.

Regarding the effect of central corneal thickness, we found only a weak, non-significant correlation between CCT and inter-device differences (r = 0.13, *p* = 0.61). The regression model revealed a theoretical inflection point at 717 µm. For the actual CCT values in our sample—ranging from 474 µm to 648 µm—the expected IOP difference between devices remained negligible (from −0.51 to −0.14 mmHg). This suggests that, while CCT is a known confounder in applanation tonometry, its impact on the relative performance of these two instruments is minimal.

This study has several methodological strengths, including its real-world clinical setting, consistent examiner technique, and appropriate statistical analysis. However, limitations should be acknowledged. The retrospective design introduces inherent selection bias, as data were collected from patients who had both tonometers used in routine practice rather than through controlled randomization. The sample size, while numerically modest, was statistically sufficient to address the primary endpoints, as determined by formal power analysis. Nevertheless, the study may not fully capture device performance in subpopulations with extreme IOP values. Further studies with larger cohorts may help validate and extend these findings. Furthermore, the study excluded patients with corneal abnormalities, limiting generalizability to those with altered biomechanics.

## 5. Conclusions

In summary, the Huvitz HT5000 digital Goldmann tonometer demonstrated excellent agreement with the traditional mechanical GAT across a wide IOP range. The minimal differences observed were both statistically and clinically insignificant, supporting the device’s interchangeability in most clinical settings. While the HT5000 offers operational advantages such as enhanced digital readout and improved ergonomics, clinicians should remain aware of its limitations, particularly its inability to register extreme IOP values and its dependence on battery power. Further prospective studies involving larger and more diverse populations, including patients with corneal pathology or undergoing IOP-lowering interventions, are warranted to confirm and extend these findings.

## Figures and Tables

**Table 1:** Demographic and clinical characteristics of the study population.

Number of patients	28	
Number of eyes	53	
Mean age (years ± SD)	62.6 ± 21.3	
Age range (years ± SD)	10–88	
Corneal thickness (μm ± SD)	549 ± 48	
Males/Females	15/13	Z = 0.38, *p* = 0.70
Mean visual acuity (LogMar ± SD)	0.3 ± 0.1	
Ocular co-morbidities (no. of eyes)	Cataract	35
	Glaucoma	25
	Dry AMD	13
	Wet AMD	5
	NPDR	5
	PDR	2
	Infectious	7
	Uveitis	5
	Other	4

SD: standard deviation; LogMAR: Logarithm of the Minimum Angle of Resolution; NPDR: Non-proliferated Diabetic retinopathy; PDR: Proliferated Diabetic retinopathy; Dry AMD: non exudative age-related macular degeneration; Wet AMD: exudative age-related macular degeneration.

**Table 2:** Clinical agreement between mGAT and dGAT based on absolute intraocular pressure (IOP) differences.

Absolute IOP Difference (mmHg)	Number of Eyes	% of Total Eyes
0	26	49.1
1	18	34.0
2	4	7.5
3	3	5.7
4	2	3.8

IOP: Intraocular pressure (IOP); mGAT: Mechanical Haag-Streit AT900 Goldmann applanation tonometer; dGAT: Digital Huvitz HT5000 applanation tonometer.

## Data Availability

The data presented in this study are available on request from the corresponding author. The data are not publicly available due to patient confidentiality and institutional restrictions.
